# Diabetes Mellitus Mortality Trends in Brazil From 2000 to 2021: An In-Depth Joinpoint Analysis

**DOI:** 10.7759/cureus.51632

**Published:** 2024-01-04

**Authors:** Billy McBenedict, Wilhelmina Hauwanga, Javier F Lizarazo, Albine Djeagou, Ifrah Akram

**Affiliations:** 1 Medicine, Hospital Universitário Antônio Pedro, Niterói, BRA; 2 Family Medicine, Faculty of Medicine, Federal University of the State of Rio de Janeiro, Rio de Janeiro, BRA; 3 General Practice, Universidad de Ciencias Médicas (UCIMED), San José, CRI; 4 General Practice, Faculty of Health Sciences, University of Buea, Buea, CMR; 5 Internal Medicine, Abbasi Shaheed Hospital, Karachi, PAK

**Keywords:** joinpoint regression, age-adjusted mortality rate, dm1, dm2, diabetes mellitus

## Abstract

Diabetes mellitus (DM) is a public health concern in Brazil, with deleterious effects on quality of life and increasing mortality rates. The prevalence of diabetes in Brazil is on the rise, and it is imperative to understand its effects on mortality rates in the last two decades in order to effectively mitigate the detrimental impact of diabetes on public health. This study aims to analyze mortality trends related to diabetes in Brazil from 2000 to 2021, encompassing both type 1 and type 2 diabetes, across sex and various age cohorts. Using joinpoint regression analysis, temporal trends in Brazil were assessed, while also incorporating findings from previous studies and considering potential influencing factors, such as government initiatives and cuts in healthcare investment. The study revealed a general upward trend in mortality rates associated with DM1 and DM2 over the study period, in both males and females, with men showing a higher AAPC (average annual percent change), which translated into significantly increased mortality difference at the end of the study. Additionally, it revealed elevated mortality values for extreme age groups in the age cohorts studied, with the exception of middle-aged cohort groups in DM2, which showed an expected higher APC (annual percent change), considering the age of highest incidence of DM2 in those age groups. This comprehensive analysis provides critical insights into the escalating impact of diabetes on mortality rates in Brazil and highlights the urgent need for healthcare strategies. It is expected that the increased prevalence of diabetes in the Brazilian population adds an additional economic burden to healthcare expenditure by the Brazilian government, further worsening the health disparities among different social groups. Unless several political decisions to reduce healthcare expenditure are reversed, greater difficulties in accessing treatments will be detrimental for vulnerable social groups in Brazil. By understanding the nuanced patterns of diabetes-related mortality, healthcare providers and policymakers can allocate resources effectively and implement tailored interventions to better address diabetes in Brazil.

## Introduction

Diabetes mellitus (DM) is considered a global public health concern that inflicts substantial pressure on healthcare systems, with an augmenting impact on mortality rates [1]. The prevalence of diabetes in Brazil is on the rise, with an estimated five million individuals currently affected by the disease. This number is expected to increase to 11 million by 2040 [1]. Therefore, the resulting impact of diabetes-related morbidity and mortality may continue to pose an economic burden worldwide, and in Brazil, unless effective healthcare measures are implemented [2], DM encompasses a spectrum of metabolic disorders marked by persistent hyperglycemia [2]. Diabetes precipitates and promotes endothelial dysfunction, vascular inflammation, arterial remodeling, atherosclerosis, dyslipidemia, and obesity as part of its pathophysiological mechanism [2]. Type 1 diabetes causes autoimmune destruction of insulin-producing cells in the pancreas and leads to a complete deficiency of insulin, and it typically emerges in childhood with sudden onset symptoms [3]. It’s caused by the interplay of genetic and environmental factors. Genetic factors include certain alleles of the HLA (human leukocyte antigens) class II genes that lead to the autoimmune destruction of pancreatic islet cells and a family history of diabetes type 1 [3]. Conversely, type 2 diabetes (T2DM) is more prevalent and has strong genetic ties along with significant links to obesity and sedentary lifestyles [3]. T2DM involves both insulin resistance and impaired secretion, resulting in a relative shortage of insulin. This form often goes undiagnosed for extended periods [3]. T2DM, being the more prevalent form, poses a greater burden on healthcare systems.

Currently, diabetes afflicts over 422 million individuals worldwide, with projections estimating a surge to 552 million by 2030 [4]. In the Brazilian context, 2017 statistics revealed that approximately 4.4% of the population was afflicted with diabetes, with corresponding disability-adjusted life years lost close to 3.3% [5]. The upward disease burden projections justify the need to monitor and study the patterns of DM in the Brazilian population. Improvements in mortality registration in Brazil now allow for a more accurate insight into diabetes-related mortality trends, which has allowed previous studies to characterize Brazil's diabetes trends [6]. The latest study conducted in Brazil provided sex and regional diabetes burden estimates in 2017, however, it didn’t take into account age and the impact of diabetes across several age cohorts; no other analysis besides it included data after 2011 [6,5]. It is possible that recent government initiatives like the "Health Has No Price" Program, first introduced in 2011, which allows pharmacies to provide insulin and oral medications for diabetics free of charge, could potentially have had a beneficial impact on mortality numbers associated with diabetes in the last decade, which highlights the importance to study data that includes trends up to 2021 in order to understand the impact of these political decisions in incidence and diabetes-related mortality, and determine efficacy of future government interventions [7]. Therefore, we conducted an in-depth analysis using joinpoint regression to better elucidate mortality rate disparities in diabetes types and sex, and explore trends across various age groups over time. By identifying key factors influencing these mortality patterns, public health officials and healthcare providers can better allocate resources and develop targeted interventions to address DM in Brazil.

## Materials and methods

Study design and data collection

We carried out a descriptive, time series study using diabetes mortality data from Brazil, from the year 2000 to 2021. Mortality data was obtained from the Brazilian Hospital Information System (DATASUS). Under the Sistema Único de Saúde (SUS), a Brazilian Unified Health System, DATASUS gathers information from all hospitalizations reimbursed by the SUS, which includes approximately 80% of the Brazilian population. The 10th edition of the International Classification of Disease (ICD 10) was used for records selection, and those coded E10 to E14 were included (which corresponds to T1DM, T2DM, malnutrition-related DM, other specified DM, and unspecified DM).

Population estimates and mortality rates

Population and demographic information of counts based on variables such as sex, age, and type of diabetes were obtained from the population estimates provided by the Brazilian Institute of Geography and Statistics (IBGE), under the Demographic and Socioeconomic Information section. We calculated the age-standardized mortality rate of diabetes for all ages. Mortality rates were expressed per 100,000 persons, and age-adjusted rates were calculated by direct standardization, using the world standard population [8].

Time series analysis

To assess temporal trends in diabetes mortality rates, we computed the average annual percentage changes (AAPCs) along with their respective 95% confidence intervals (CIs) employing joinpoint regression [8]. The AAPC was derived as a geometrically weighted mean of different annual percentage change (APC) values acquired from the regression analysis. Joinpoint Regression Program (Version 5.0.2. May 2023) is a trend analysis software developed by the Statistical Research and Applications Branch of the National Cancer Institute (Bethesda, MD, United States), for the analysis of data from the Surveillance Epidemiology and End Results Program (SEER). Weighted Bayesian Information Criteria were used to test for the level of significance and determine the best-fitting combination of line segments and joinpoints [8]. This examination aimed to investigate variations in diabetes disease mortality trends across categories such as age and sex.

## Results

Trends in mortality related to all types of DM from 2000 to 2021

All Types of Diabetes Combined Based on Sex

**Figure 1:**
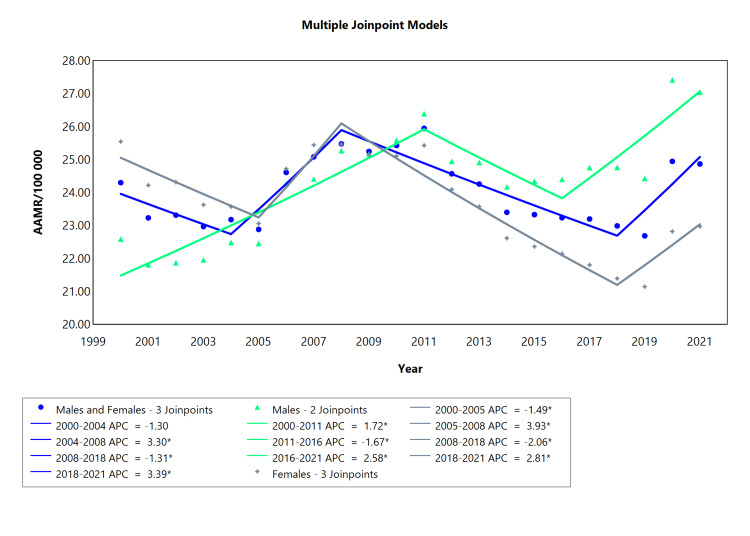
Diabetes AAMR trend joinpoint analysis for the variable sex during the period 2000-2021. *Significant at P < 0.05 level AAMR = age-adjusted mortality rate; APC = annual percent change

The trend in mortality related to all types of diabetes was characterized by three joinpoints for females, while the trend in males had two joinpoints (Figure 1, Table 1). There was a significant rise in diabetes-related mortality for both sexes from 2004 to 2008 (APC = 3.30; CI: 1.56 to 5.55), a drop was observed from 2008 to 2018 (APC = -1.31; CI: -2.86 to -0.89) and an increase from 2018 to 2021 (APC = 3.39; CI: 0.62 to 7.06). The mortality rate in females followed a similar trend, with an initial decrease from 2000 to 2005 (APC = -1.49; CI: -3.97 to -0.52), followed by an upward deviation seen in the period from 2005 to 2008 (APC = 3.93; CI: 1.59 to 5.17), then a drop from 2008 to 2018 (APC = -2.06; CI: -3.00 to -1.71) and finally an upward trend from 2018 to 2021 (APC = 2.81; CI: 0.31 to 6.35). The trend in males differed with the number of joinpoints and periods of change. The mortality rate increased significantly for males from 2000 to 2011 (APC = 1.72; CI: 1.21 to 2.57), followed by a decrease from 2011 to 2016 (APC = -1.67; CI: -4.46 to -0.02) and a subsequent rise from 2016 to 2021 (APC = 2.58; CI: 0.95 to 6.85).

**Table 1:** Trend in mortality related to all types of diabetes amongst Brazilians aged 10 and above from 2000 to 2021. *Significant at P < 0.05 level AAPC = average annual percent change; APC = annual percent change

Sex	Period (year)	APC (95% CI)	AAPC (95% CI)
Males and Females	2000 to 2004	-1.30 (-47 to 0.35)	0.22 (-0.07 to 0.48)
Males and Females	2004 to 2008	3.30* (1.56 to 5.55)	
Males and Females	2008 to 2018	-1.31* (-2.86 to -0.89)	
Males and Females	2018 to 2021	3.39* (0.62 to 7.06)	
Males	2000 to 2011	1.72* (1.21 to 2.57)	1.11* (0.82 to 1.42)
Males	2011 to 2016	-1.67* (-4.46 to -0.02)	
Males	2016 to 2021	2.58* (0.95 to 6.85)	
Females	2000 to 2005	-1.49* (-3.97 to -0.52)	-0.40* (-0.65 to -0.19)
Females	2005 to 2008	3.93* (1.59 to 5.17)	
Females	2008 to 2018	-2.06* (-3.00 to -1.71)	
Females	2018 to 2021	2.81* (0.3 to 6.35)	

The mortality rate in females followed a similar trend, with an initial decrease from 2000 to 2005 (APC = -1.49; CI: -3.97 to -0.52), followed by an upward deviation seen in the period from 2005 to 2008 (APC = 3.93; CI: 1.59 to 5.17), then a decrease from 2008 to 2018 (APC = -2.06; CI: -3.00 to -1.71) and finally an increment from 2018 to 2021 (APC = 2.81; CI: 0.31 to 6.35). The trend in males differed with the number of joinpoints and periods of change. The mortality rate rose significantly for males from 2000 to 2011 (APC = 1.72; CI: 1.21 to 2.57), followed by a decrease from 2011 to 2016 (APC = -1.67; CI: -4.46 to -0.02) and an increase from 2016 to 2021 (APC = 2.58; CI: 0.95 to 6.85).

Trends in mortality related to type 1 diabetes from 2000 to 2021

Type 1 Diabetes Based on Sex

**Figure 2:**
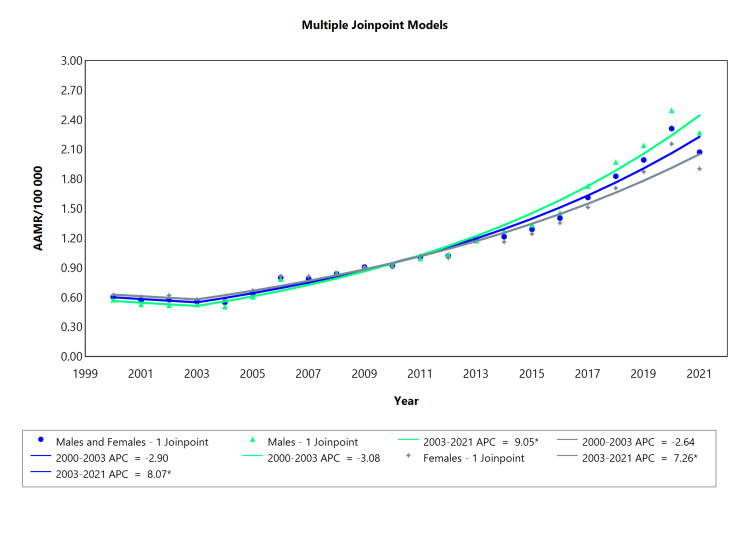
Type 1 diabetes AAMR trend joinpoint analysis for the variable sex during the period of 2000-2021. *Significant at P < 0.05 level AAMR = age-adjusted mortality rate; APC = annual percent change

The study was divided into two segments. The first one spanned from 2000 to 2003, composed of three cohorts, male and women combined, male, and women. The second segment spanned from 2003 to 2021 and had the same subdivisions as the first one. There was a statistically significant increase in the AAPC in type 1 diabetes in all subgroups, males: 7.2281 CI (6.2879 to 8.8267), females: 5.7892 CI (4.9255 to 7.0377), and both sexes: 6.4329 CI (5.5600 to 7.8274). The mortality rate associated with T1DM displayed a consistent increase during the primary endpoints 2003-2021 as presented in Figure 2. There was one segment in the cohort of males and females from 2003 to 2021 (APC = 8.07; CI: 7.4 to 10.9). Similarly, a significant positive trend was observed from 2003 to 2021 for males within the second segment (APC = 9.05; CI: 8.28 to 12.33) as well as for females within the second segment (APC = 7.26; CI: 6.65 to 10.02). Although there was a reduction in mortality rates of type 1 diabetes between 2000 and 2003, this decline was not considered statistically significant, i.e. APC = -2.9 and CI = -14.4 to 7.23 in both sexes.

There was a positive correlation between T1DM and age-adjusted mortality rates (AAMRs) with an almost fourfold rise in the period between 2003 and 2021 in the male subgroup. Females were also affected, albeit with a less pronounced association. Both subgroups showed statistically significant results in this period (Table 2).

**Table 2:** Trend in mortality related to type 1 diabetes amongst Brazilians from 2000 to 2021. *Significant at P < 0.05 level AAPC = average annual percent change; APC = annual percent change

Sex	Period (year)	APC (95% CI)	AAPC (95% CI)
Males and Females	2000-2003	-2.90 (-14.5 to 7.24)	6.43* (5.56 to 7.83)
Males and Females	2003-2021	8.07* (7.41 to 10.92)	
Males	2000-2003	-3.08 (15.80 to 8.30)	7.23* (6.23 to 8.83)
Males	2003-2021	9.05* (8.28 to 12.34)	
Females	2000-2003	-2.64 (-13.65 to 6.43)	5.79* (4.93 to 7.04)
Females	2003-2021	7.26* (6.65 to 10.02)	

Type 1 Diabetes Based on Age Range

**Figure 3:**
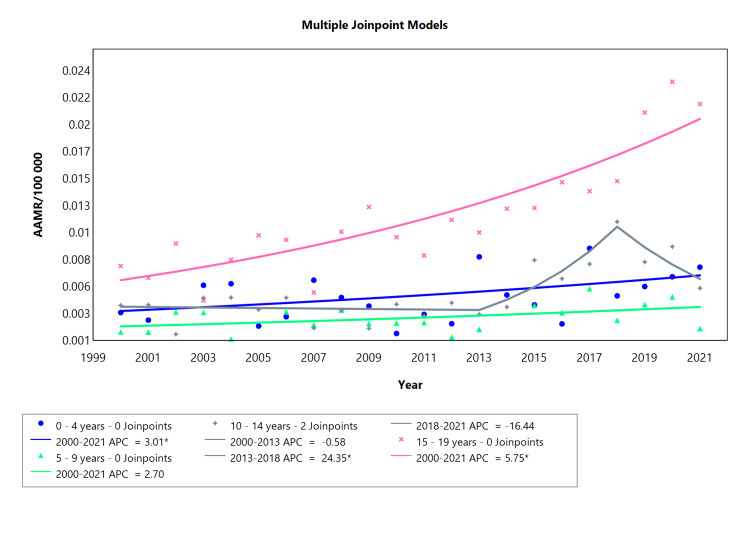
Diabetes type 1 AAMR trend joinpoint analysis for the variable age (children) during the period 2000-2021. *Significant at P < 0.05 level AAMR = age-adjusted mortality rate; APC = annual percent change

In the analysis of type 1 diabetes by age groups younger than 20 years of both sexes, the data was divided into four cohorts (Table 3). The first group i.e., 0-4 years old showed 0 joinpoints with an APC of 3.01 and a similar AAPC of 3.01 for the whole study period. The 5-9 years category did not manifest any significant results. The cluster of 10-14-year-olds presented the most dynamic mortality rates of type 1 diabetes, declining initially between 2000 and 2013 (APC = -0.58), then a rapid surge from 2013 up to 2018 (APC = 24.35) and eventually a steady fall in mortality cases from 2018 to 2021 (APC = -16.44). Teenagers aged 15-19 years experienced the highest incidence of mortality from type 1 diabetes, gradually rising from 2000 to 2021 with APC and AAPC values of 5.75 as denoted by Figure 3.

**Table 3:** Trends in mortality related to type 1 diabetes amongst Brazilians younger than 20 years from 2000 to 2021. *Significant at P < 0.05 level AAPC = average annual percent change; APC = annual percent change

Age range (years)	Period (year)	APC (95% CI)	AAPC (95% CI)
0-4 years	2000-2021	3.01* (0.06 to 6.79)	3.01* (0.06 to 6.79)
5-9 years	2000-2021	2.70 (-1.27 to 7.67)	2.70 (-1.27 to 7.67)
10-14 years	2000-2013	-0.58 (-10.45 to 3.89)	2.29 (-0.59 to 4.80)
10-14 years	2013-2018	24.35 (10.04 to 53.99)	
10-14 years	2018-2021	-16.44 (-41.75 to 0.82)	
15-19 years	2000-2021	5.7540* (4.27 to 7.78)	5.75* (4.27 to 7.78)

**Figure 4:**
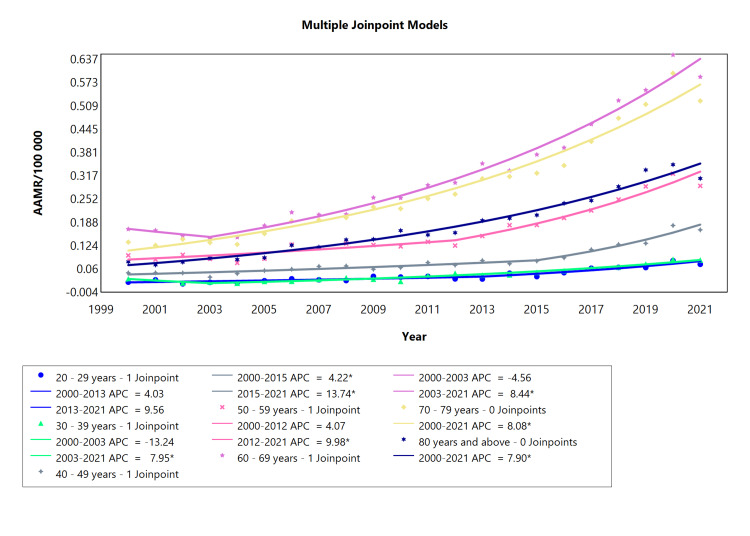
Diabetes type 1 AAMR trend joinpoint analysis for the variable age (adults) during the period 2000-2021. *Significant at P < 0.05 level AAMR = age-adjusted mortality rate; APC = annual percent change

The data of T1DM was also analyzed for adults (males and females combined) of all age groups from 20 years to 80 years and above for the study period 2000-2021 (Table 4). The 20-29 years did not show significant APC but the AAPC of this group was significant (AAPC = 6.1040). The category of adults aged 30-39 years showed 1 joinpoint with an APC of -13.24 between 2000 and 2003, a significant APC value of 7.95 from 2003 to 2021, and AAPC of 4.63 for the whole period (Figure 4). The 40-49 years group also showed 1 joinpoint, APC = 4.22 during 2000-2015, APC of 13.74 in the years 2015-2021, likewise a significant AAPC of 6.85 for the whole segment. The 50-59 year-olds displayed 1 joinpoint where type 1 diabetes caused the highest number of deaths in the period 2012-2021 (APC = 9.98 and AAPC=6.56). The cohort of the 60-69 year group manifested the most notable results with a declining mortality trend in the initial years, APC = -4.56 in 2000-2003, and a rapidly rising AAMR from 2003 up to 2021 (APC = 8.44). The last two groups i.e., 70-79 years and 80 years and above, both presented 0 joinpoints, with APC and likewise AAPC values of 8.08 and 7.90 respectively.

**Table 4:** Trends in mortality related to type 1 diabetes amongst Brazilians older than 20 years from 2000 to 2021. *Significant at P < 0.05 level AAPC = average annual percent change; APC = annual percent change

Age range (years)	Period (year)	APC (95% CI)	AAPC (95% CI)
20-29 years	2000-2013	4.03 (-12.08 to 33.47)	6.10* (4.40 to 9.09)
20-29 years	2013-2021	9.56 (-3.39 to 26.88)	
30-39 years	2000-2003	-13.24 (-27.99 to 0.20)	4.63* (3.71 to 6.19)
30-39 years	2003-2021	7.95* (7.12 to 9.47)	
40-49 years	2000-2015	4.22* (0.44 to 5.97)	6.86* (5.76 to 8.05)
40-49 years	2015-2021	13.74* (9.04 to 28.44)	
50-59 years	2000-2012	4.07 (-4.39 to 6.29)	6.57* (5.53 to 7.75)
50-59 years	2012-2021	9.98* (7.72 to 18.50)	
60-69 years	2000-2003	-4.56 (-16.22 to 5.99)	6.48* (5.70 to 7.72)
60-69 years	2003-2021	8.44* (7.88 to 9.74)	
70-79 years	2000-2021	8.08* (7.44 to 8.96)	8.08* (7.44 to 8.96)
80 years and above	2000-2021	7.90* (7.22 to 8.87)	7.90* (7.22 to 8.87)

Trends in mortality related to type 2 diabetes from 2000 to 2021

Type 2 Diabetes Based on Sex

**Figure 5:**
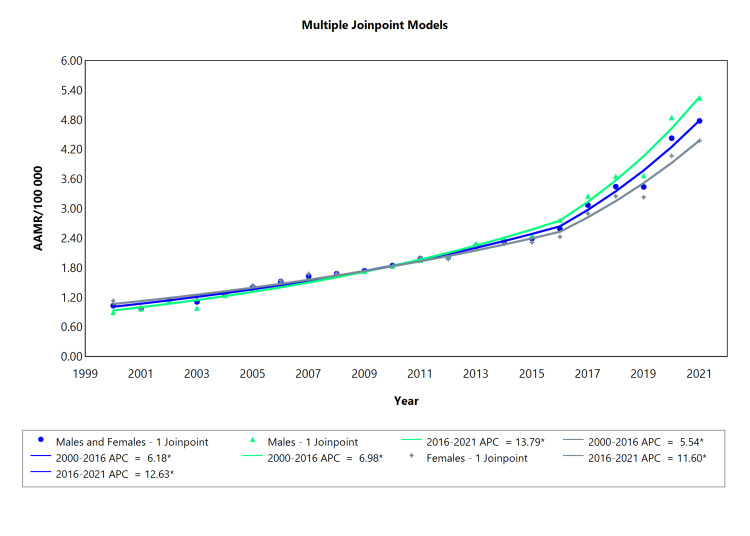
Type 2 diabetes AAMR trend joinpoint analysis for the variable sex during the period of 2000-2021. *Significant at P < 0.05 level AAMR = age-adjusted mortality rate; APC = annual percent change

The mortality rate related to type 2 diabetes increased during the period 2000 to 2021 for both males and females, but this increment was more profound for males compared to females, as evidenced by the significant AAPC of 8.56 and 6.95 in males and females, respectively. There were two segments observed overall and for each of the two categories of sex, with statistically significant APCs, as presented in Table 5. A gradual increase was observed from 2000 to 2016 with significant APC values of 6.18l, 6.98 for males, and 5.54 for females. There was a rapid increase in the mortality rates of T2DM from 2016 to 2021, with APC values of 12.63 for all sex categories, 13.79 for males, and 11.60 for females. The rate of increase was higher for males compared to females during both periods (Figure 5). The mortality rate related to type 2 diabetes increased during the period 2000 to 2021 for both males and females (Figure 3), but this increase was more notable in males compared to females, as evidenced by the significant AAPC of 8.56 and 6.95 in males and females, respectively.

**Table 5:** Trends in mortality related to type 2 diabetes amongst Brazilians from 2000 to 2021. *Significant at P < 0.05 level AAPC = average annual percent change; APC = annual percent change

Sex	Period (year)	APC (95% CI)	AAPC (95% CI)
Males and Females	2000-2016	6.18* (5.36 to 6.90)	7.68* (7.28 to 8.17)
Males and Females	2016-2021	12.63* (10.36 to 17.50)	
Males	2000-2016	6.98* (6.04 to 7.79)	8.56* (8.11 to 9.12)
Males	2016-2021	13.79* (11.38 to 19.00)	
Females	2000-2016	5.54* (4.46 to 6.33)	6.95* (6.45 to 7.52)
Females	2016-2021	11.60* (8.90 to 18.81)	

The average annual mortality rate increased from 1.03 deaths per 100,000 in 2000 to 4.78 deaths per 100,000 of the population from both males and females. The mortality rate increased from an average of 0.90 deaths per 100,000 to 5.25 deaths per 100,000 for males and from an average of 1.13 deaths per 100,000 to 4.38 deaths per 100,000 for females. There were two segments observed overall and for each of the two categories of sex, with statistically significant APCs, as presented in Table 5. A gradual increase was observed from 2000 to 2016 with significant APC values of 6.18 overall, 6.98 for males, and 5.54 for females. There was a rapid increase in the mortality rate related to T2DM from 2016 to 2021, with APC values of 12.63 for all sex categories, 13.79 for males, and 11.60 for females. The rate of increase was higher for males compared to females during both periods.

Type 2 Diabetes Based on Age Range

**Figure 6:**
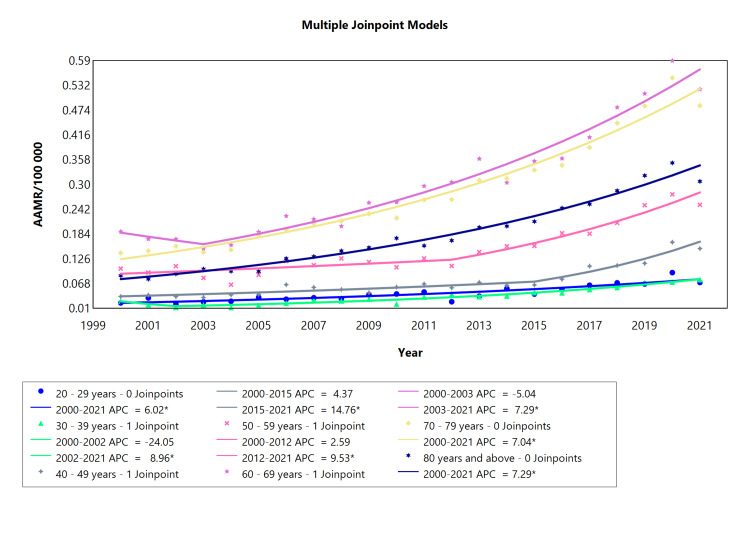
Type 2 diabetes AAMR trend joinpoint analysis for the variable age (adults) during the period of 2000-2021. *Significant at P < 0.05 level AAMR = age-adjusted mortality rate; APC = annual percent change

Regarding diabetes type 2 age subgroups, all cohorts were subdivided into two segments with the exception of the 20-29 years, 70-79 years, and 80 years and above groups. All these categories showed statistically significant increases in APC. When analyzing the full range of each cohort, the highest values obtained for APC in T2DM mortality were consistent with the middle age (40-49 years) from 2015 to 2021 (APC = 14.76) with a second peak in the succeeding group: the 50-59-year-old between 2012 and 2021 (APC = 9.53). The mortality associated with AAPC was lowest in the 30-39 years old with a value of 5.27 and highest in the eldest population (AAPC = 7.28) (Table 6, Figure 6).

**Table 6:** Trends in mortality related to type 2 diabetes amongst Brazilians older than 20 years from 2000 to 2021. *Significant at P < 0.05 level AAPC = average annual percent change; APC = annual percent change

Age range (years)	Period (year)	APC (95% CI)	AAPC (95% CI)
20-29 years	2000-2021	6.0194* (4.7989 to 7.6444)	6.0194* (4.7989 to 7.6444)
30-39 years	2000-2002	-24.0521 (-34.9565 to 6.0592)	5.2761* (4.0848 to 8.1301)
30-39 years	2002-2021	8.9577* (7.8489 to 12.4399)	
40-49 years	2000-2015	4.3733 (-1.1013 to 6.4300)	7.2399* (5.8543 to 8.6972)
40-49 years	2015-2021	14.7555* (9.3133 to 31.4616)	
50-59 years	2000-2012	2.5921 (-8.4723 to 5.2333)	5.5100* (4.2245 to 6.9758)
50-59 years	2012-2021	9.5300* (6.6560 to 21.4691)	
60-69 years	2000-2003	-5.0366 (-17.2957 to 6.0807)	5.4361* (4.5893 to 6.9352)
60-69 years	2003-2021	7.2906* (6.5421 to 10.5460)	
70-79 years	2000-2021	7.0428* (6.4399 to 7.8796)	7.0428* (6.4399 to 7.8796)
80 years and above	2000-2021	7.2872* (6.7036 to 8.1032)	7.2872* (6.7036 to 8.1032)

## Discussion

This study aimed to evaluate the trends in mortality related to type 1, type 2, and all types of diabetes in Brazil from 2000 to 2021. The analysis was done by sex and age groups. As per the World Health Organization, diabetes caused 1.5 million deaths worldwide in 2019, and another 460,000 deaths due to diabetes-related kidney disease. According to our results, the rate of death related to diabetes types 1 and 2 increased significantly during the period 2000 to 2021. However, when considering all types of diabetes, there were variations in trend, with periods of increase and decrease, but the later part of the study period was characterized by an increase in mortality for all. Diabetes decreases life expectancy [9], and patients with diabetes have been reported to have 2-3 times increased risk of death compared to those without diabetes [10].

The increased mortality rate could be explained by the increased incidence and prevalence of diabetes in low- and middle-income countries (LMICs), which has been reported previously in Latin America [11]. Rapid economic development, urbanization, and increased sedentarism have been a significant contributor to the upsurge in diabetes incidence and prevalence [12]. The growth in incidence, prevalence, and mortality can also be partly due to the effect of the modifications in the diagnostic criteria [13]. As opposed to our findings, Schmidt and colleagues reported a rather decreasing mortality rate while analyzing the trends in mortality due to diabetes in Brazil from 1996 to 2011 [6]. This difference in findings could be explained by the fact that they included only those aged 30 to 69, meanwhile we included all ages. The difference in trend observed in our study could have been influenced by the age extremes. Worldwide, AAMRs for all types of diabetes have not seen a steady pattern.

Type 1 diabetes based on sex

Our study showed an increasing trend for DM-related mortality for both Brazilian men and women, but the rate of increase was significantly higher for men than women. In 2016, a study analyzed the sex differences in DM-related mortality in Brazil from 1980 to 2012 and they also observed an upward mortality trend for both males and females, with an initial female predominance and a reversal in predominance from 2011 onwards [14]. In 2021, Florencio and colleagues compared the hospitalization rate and mortality rate related to DM between 2008 and 2019 in Brazil, and similar to our findings, they reported an increase, which was higher for men than women [15]. This increased mortality rate amongst men could be explained by the fact that men are more likely to have diabetes than women as proposed by an analysis in India and in Spain [16,17], and thus more likely to die from its complications. This difference in diabetes risk has been attributed to the fact that men tend to have a higher amount of visceral fat than women [18]. When compared to women with similar BMIs, men were found to have higher fat storage and insulin resistance [19]. Moreover, women are more careful with their health compared to men, and they are more likely to seek medical help [20].

Contrary to our findings, other studies in China reported higher diabetes-related mortality rates among women than men [21]. In a systematic review with meta-analysis published in 2019, a higher mortality hazard ratio was observed for women compared to men [22]. One of the limitations stated in this study was the high rate of self-reported diabetic status in most of the studies included. It was previously found that the sensitivity of self-reported diabetes is less for women than for men [23], therefore if fewer women are likely to report their diabetes status correctly, it is possible that those identified by self-reporting are the most severe ones who are obviously more at risk of death. It is worth noting that these studies were performed in other countries with different ethnic and racial distributions. Ethnicity and race have been found to influence the risk of and progression of disease [24], which could explain why the pattern observed in the Brazilian population is different from that observed elsewhere. The difference could also be explained by a better adherence to treatment by Brazilian women [25], which might not be the case in other settings.

Type 1 diabetes based on age

The incidence of type 1 diabetes is increasing worldwide annually (3-5%), with rates doubling every 20 years, as stated by research in America [26]. Our investigation found an analogous upward trend in the mortality rates for all age strata between 2000 and 2021, with the only exception being the age group 10-14 years, which initially displayed a stable mortality rate from 2000 to 2013, then a rapid rise between 2013 and 2018 and eventually a sharp decline from 2018 to 2021. Mortality rates were highest in the cohort of 60-69-year-olds, followed by the 70-79-year age group, pointing towards the fatal long-term complications of diabetes such as nephropathy, neuropathy, ischemic heart diseases, and peripheral vascular disease among many others [27]. A cross-sectional study done previously in Brazil in 2015 showed contrary results with a moderately declining pattern of diabetes in the 30-69 years age group during its study period spanning from 1996 to 2011 [6]. Among Brazilian populations younger than 20 years, the highest rates of mortality were recorded in adolescents aged 15-19 years, indicating the lethal outcomes of insulin-dependent DM like diabetic ketoacidosis, cerebral edema, cardiovascular complications, and renal failure. To prevent type 1 diabetes, several measures must be taken such as the elimination of causative environmental factors, the use of mitigating factors like vitamin D, breastmilk and poly-unsaturated fatty acids, the reverse of epigenetic modifications, and the use of autoantigen-based immunotherapy [28]. Mortality rates in high-income countries have been steadily decreasing due to better improvements in healthcare access and diabetes management, but this improvement was less marked and in the case of Brazil even worsened in impoverished countries with lower healthcare expenditure [29,30].

Type 2 diabetes based on sex

Mortality rates associated with type 2 diabetes had a steady increase in the first segment that covered the period between 2000 and 2016. There was an upward shift in the trend during the second segment that spanned from 2016 to 2021 that affected both sexes. The accelerated rise in mortality associated with type 2 diabetes observed in the second segment could be partly explained by reduced healthcare access due to government austerity measures implemented in 2016, which included withdrawing from the key social protection plan “Brazil Without Extreme Poverty”, and reductions in more than 70 social and healthcare programs made for vulnerable populations [31]. Although it is expected that this change had an impact on the long term, immediate reduced access to medications could’ve precipitated acute complications of type 2 diabetes (for example, hyperosmolar hyperglycemic state) or other organ dysfunctions (i.e., diabetic nephropathy), augmenting mortality rates on the short term. More studies are needed to elucidate if an increased incidence of acute complications of type 2 diabetes correlates with this time period. A study evaluating DM2 mortality rates in several countries found that while lower-income countries have had an increase in AAMRs, higher-income countries have undergone a reduction in AAMR associated with type 2 diabetes, mainly attributed to lower rates of microvascular and macrovascular complications. This same study characterized Brazil as a middle-income country with a steady upward trend in AAMRs, with a decrease in all-cause mortality rates including acute complications, possibly explained by better intra-hospital management of these complications, even if there was a rise in hospitalizations from 2008 to 2019 due to DM2 [32].

There was a slight disparity in mortality rates between males and females at the end of the study. Males had minimally higher mortality rates compared to females after the 21-year period. Mortality rates were pretty similar at the beginning of the study, but around 2012 onwards, a slight deviation occurred between both sexes, shown by the difference in APC values of 13.79 (CI 11.38-19.00) and 11.60* (8.90-18.81) for males and females, respectively. This is consistent with other studies conducted in Brazil that showed men to have higher mortality rates compared to females with DM2, which also highlighted the shift (males now having higher mortality rates associated with DM2 than females) in 2011 [5]. The fact that women have been classically known for having a greater perception of health problems and are more likely to consult for healthcare concerns, tied with the greater access to healthcare in Brazil during the last decade, could’ve played a key factor in mortality rate disparities between both sexes at the end of the study [7,20]. However, other studies in Brazil yielded contradictory results, demonstrating women to have consistently higher mortality than men, which underscores the need for additional research to determine if there was really a paradigm shift occurring around 2011-2012 regarding the mortality rate annual increase based on sex [6].

Type 2 diabetes based on age

Age cohorts in T2DM rose constantly in AAMR across all age groups, with a bimodal distribution in terms of average APC increase, with the exception of the 40-49-year-old subgroup, which showed comparatively higher than average increase in mortality rates. Although epidemiological studies previously done in Brazil showed that T2DM incidence was relatively low in the 30-34 (200 cases per 100,000) and 35-40 (nearing 300 cases per 100,000), this population has the greatest increase in myocardial infarction (14-fold HR 14.0, 95% CI 6.2-31.4) comparatively with DM diagnosed in older groups (4-fold increase HR 3.7, p < 0.001), possibly due to the impact of long term macrovascular and microvascular damage [33,5]. This highlights the importance of screening programs for T2DM in Brazil, which could diminish the associated comorbidities in T2DM, and with the subsequent reduction in T2DM-associated mortality rates. Other age groups that showed greater mortality rates associated with T2DM were the elderly 80+ and young population. However, due to the almost negligible prevalence of T2DM in the younger population, the impact on resources and overall mortality rate was greater in the 40+ population. Current recommendations by the 2021 US Preventive Services Task Force (USPSTF) are to screen anyone above 35 years of age with overweight or diabetes, and the American Diabetes Association (ADA) 2023 guidelines recommend screening everyone above 35 [34]. Such measures implemented at a national level in Brazil would likely reduce mortality rates in the long term. However, even if additional measures are implemented state-wise, it is critical to improve healthcare literacy to improve overall outcomes and adherence to treatment [35].

Strengths and limitations of the study

Strengths of this study include the availability of data for a longer period (21 years) which allows the detection of the slightest changes in mortality patterns. Also, despite the existence of similar studies in Brazil, this is the first to our knowledge to have analyzed DM-related mortality trends in Brazil until 2021, which is quite recent. Thanks to this study, we were able to identify the rapid increase in mortality during recent years, requiring urgent actions. Using data from the mortality information system which is a national database is quite representative of the Brazilian population. This avoids the bias often observed in other study types, which could be less representative and difficult to generalize to the entire nation. Reliance on objective data recorded in death certificates rather than self-reported data on disease status which could under- or over-estimate the rates observed.

The limitations of the study are linked to the Brazilian Mortality Information system, though being universal, the coverage of deaths and quality of information on causes of death is disproportionate across states and varies with time [36]. Another potential source of weakness of our study could be related to the quality and accuracy of data, as unreported cases could result in incomplete data and the assignment of inappropriate ICD-10 codes to the mortality cases. However, a considerable improvement has been observed with an increase in completeness of death counts from 80% in 1980-1991 to 95% in 2000-2010, and a decrease in the proportion of ill-defined causes of death by about 53% during the same period [37].

## Conclusions

Mortality rates for all types of diabetes are escalating rapidly in Brazil, with T2DM showing the fastest growth at a 7.68* (7.28 to 8.17) AAPC for both men and women, particularly accentuated in men who manifested the fastest increase in AAPC. Although there was an upward inflection point in 2015 potentially linked to the latent impact of austerity measures implemented by the government around 2011, additional investigations are warranted to elucidate the driving factors behind this observed change in pattern. Mortality rates also rose consistently across all age cohorts, with the elderly population showing the fastest rise in APC in both T1DM [7.2872* (6.7036 to 8.1032)] and T2DM. In regards to T2DM, mortality rates also experienced higher APC in middle-aged groups, consistent with the population with the highest incidence of acute T2DM complications, mainly cardiovascular. This study is in line with other studies worldwide that characterized Brazil’s mortality rates as increasing for both T2DM and T1DM, with the added value of providing more recent data and highlighting points of upward deviation in the trends that should be alarming to healthcare authorities in Brazil. Although this study could contribute to strengthening public health policies to improve the prevention and management of diabetes, we recommend further studies to better determine the etiologies behind this accelerated rise in mortality rates in Brazil during the last decade.
